# From *Coxiella burnetii* Infection to Pregnancy Complications: Key Role of the Immune Response of Placental Cells

**DOI:** 10.3390/pathogens10050627

**Published:** 2021-05-19

**Authors:** Sandra Madariaga Zarza, Soraya Mezouar, Jean-Louis Mege

**Affiliations:** 1 Aix-Marseille University, MEPHI, IRD, APHM, 13005 Marseille, France; szarza90@gmail.com (S.M.Z.); jean-louis.mege@univ-amu.fr (J.-L.M.); 2 Institute Hospitalo-Universitaire-Méditerranée Infection, 13005 Marseille, France; 3 Unité Fonctionnelle Immunologie, Assistance Publique Hôpitaux de Marseille, 13005 Marseille, France

**Keywords:** *Coxiella burnetii*, pregnancy, trophoblasts, macrophages, dendritic cells, mast cells

## Abstract

The infection of pregnant animals and women by *Coxiella burnetii*, an intracellular bacterium, compromises both maternal health and foetal development. The placenta is targeted by *C. burnetii,* as demonstrated by bacteriological and histological evidence. It now appears that placental strains of *C.* *burnetii* are highly virulent compared to reference strains and that placental injury involves different types of placental cells. Trophoblasts, the major placental cells, are largely infected by C. *burnetii* and may represent a replicating niche for the bacteria. The placenta also contains numerous immune cells, including macrophages, dendritic cells, and mast cells. Placental macrophages are infected and activated by *C. burnetii* in an unusual way of M1 polarisation associated with bacterial elimination. Placental mast cells eliminate bacteria through a mechanism including the release of extracellular actin filaments and antimicrobial peptides. In contrast, *C. burnetii* impairs the maturation of decidual dendritic cells, favouring bacterial pathogenicity. Our aim is to review *C. burnetii* infections of human placentas, paying special attention to both the action and function of the different cell types, immune cells, and trophoblasts targeted by *C. burnetii* in relation to foetal injury.

## 1. Introduction 

*Coxiella burnetii* is a Gram-negative bacterium that infects numerous animals, including mammals, birds, and arthropods [1]. While the infection is inapparent in certain species, humans develop Q fever, which is a zoonosis with worldwide distribution. The main *C. burnetii* reservoirs’ are sheep, goats, and cattle, which are sources of human transmission [2]. The transmission pathway for humans is commonly respiratory from contaminated aerosols mainly during the delivery of abortion of infected animals [2,3]. The clinical presentation of Q fever [4] is asymptomatic in about 50% of infected people, whether acute or persistent. The manifestations of acute Q fever are atypical pneumonia, hepatitis, or flu-like self-limiting disease. In immunocompromised situations such as cancer or corticosteroid treatment, *C. burnetii* infection may become persistent and result in life-threatening endocarditis [5]. 

Pregnancy is an extraordinary situation in which the mother’s immune system must tolerate a semi-allograft, the foetus. Consequently, this environment of immunosuppression secondary to pregnancy causes increased sensitivity to certain virus, parasites [6], or bacteria (such as *Brucella abortus* or *C. burnetii)* [7], impairing pathogen clearance and increasing disease severity [8]. Indeed, *C. burnetii* infection in pregnant goats leads to abortions, and Q fever during pregnancy presents important risks for both the mother and the foetus [5]. The placenta is a nutrient tissue at the maternal–foetal interface involved in both foetal growth and foetal tolerance. It is rich in a large number of immune cells, including uterine natural killer cells (NK cells), macrophages, lymphocytes, dendritic cells (DCs), and mast cells. 

This review first summarises the impact of Q fever on pregnancy and the emergence of obstetrical complications. Then, this study proposes analysing the infection of major innate immune placental cells by *C. burnetii* and the dysregulation of the placental immune response, leading to the pathophysiology of Q fever in pregnancy.

## 2. Natural History of *C. burnetii* Infection in Pregnancy

In 1958, Syrucek et al. isolated *C. burnetii* organisms from aborted human placentas [9]. It is now known that placental infection by *C. burnetii* mostly occurs during the first and second trimesters of pregnancy [10]. Women who are infected during pregnancy are at risk of miscarriage, stillbirth, pre-term delivery, low infant birth weight, and foetal death or malformations (omphalocele, hypospadias, Potter syndrome, congenital hydronephrosis, and syndactyly) and growth retardation [2,11]. It has been shown that women who contract acute Q fever just before pregnancy do not present an increased risk of abortion or premature delivery. In contrast, infection that occurs during the first trimester of pregnancy leads to abortion and during the second trimester is more likely to result in prematurity [12]. *C. burnetii* infection in pregnant women increases the risk of maternal chronic Q fever endocarditis [13,14]. Q fever during a first pregnancy favours spontaneous abortions of future pregnancies [15], suggesting Q fever reactivation during latter pregnancies [12]. In women, a silenced immune response and the production of antibodies have been reported in response to infection [16].

In recent decades, outbreaks of Q fever have been observed around the world, including in the United Kingdom in 1989, France in 1998, Germany in 2005, and the Netherlands in 2007 [17]. Seroprevalence studies in pregnant women show highly variable rates in areas of endemicity: 0.15% in southeastern France, 3.8% in Canada, and 4.6% in the United Kingdom. In Denmark, a seroprevalence rate of up to 47% was reported in pregnant women who were occupationally exposed to livestock versus 4.8% in unexposed women [2]. More specifically, a seroprevalence of acute Q fever of 1.2% was found among women who experienced spontaneous abortion in the first semester of pregnancy in Denmark, with a significant proportion of asymptomatic patients [18]. In France, the seroprevalence in women who experienced spontaneous abortion was 0.27% [18,19]. 

*C. burnetii* infection is particularly deleterious in goats. *C. burnetii* infection is responsible for metritis, abortion, stillbirth and the delivery of weak offspring, which are the most frequent clinical signs of disease [20]. The placenta of goats is very rich in bacteria, and it is often through the aerosol route that farmers are contaminated. *C. burnetii* organisms are present in different organs such as the liver, spleen, lung [5,21,22], kidney, heart [5,21], and muscles [21] of goat foetuses and their mothers. However, to date, the vertical transmission of infection remains unclear. Bacteria are found in the stomach of the foetus, indicating that they contaminate amniotic liquid [22,23]. Alternatively, the inhalation of contaminated aerosols during parturition or lactation may be another source of contamination [1,24]. 

## 3. *Coxiella burnetii*

Several *C. burnetii* strains isolated from animals or humans including patients with acute or persistent focalised Q fever have been used for research [25]. However, the Nine Mile strain, isolated from the *Dermacentor andersoni* tick in Montana in 1938, is to date the strain mostly used in host–pathogen studies [26]. The lipopolysaccharide (LPS) structure, plasmid, and genotype have been commonly related to the different disease manifestations [25,27], cytopathic effects in cell cultures [25], and immune response [28,29,30,31]. The repeated cultures of *C. burnetii* Nile Mile strain result in a truncated LPS (O-antigen modification), which is associated with virulence decrease. This transition from phase I LPS to phase II LPS is related to a ≈26 Kb chromosomal deletion of *C. burnetii* genome [32]. Despite their frequent use in studies, phase II *C. burnetii* are not suitable for pathophysiopathological studies.

Generally speaking, placental *C. burnetii* isolates appear to be more virulent than other isolates. It has been proposed that the virulence found in the different strains could be due to the variation of LPS but also to the genomic content. Correlations have been found between *C. burnetii* genome variations or LPS chemotype and clinical presentations of Q fever [33,34]. The *C. burnetii* RSA493 strain genome was the first strain sequenced in 2003, which led to significant progress in understanding of *C. burnetii* pathogenicity [35]. The genome contains a 1,995,275 bp chromosome and a QpH1 plasmid with 37,393 bp. *C. burnetii* virulence appears to be correlated with the expression of certain plasmids. Four different plasmids have been identified among *C. burnetii* isolates, including QpH1 (Nine Mile strain), QpRS (Priscilla strain), QpDG (wild rodents), and QpDV [28]. *C. burnetii* Nine Mile strain presents the plasmid QpH1, MST16, and GGI [28]. It was reported that *C. burnetti* Nine Mile strain with QpH1 plasmid is the cause of severe *C. burnetii* infection in a guinea pig model [27,29]. Moreover, QpRS and QpDG plasmids have been associated with moderate infection and lack of virulence, respectively [27,29]. Three of the plasmids identified among *C. burnetii* isolates have been found in placental isolates from France and Spain (QpH1, QpRS, and QpDV) [28]. Obstetric complications are likely to be related to the QpDV plasmid [28,36]. Indeed, this plasmid has been found in three of six placentas and in placental bacterial strains from abortive women [28,37]. However, the presence of the QpDV plasmid has also been reported in a healthy woman, and other placental strains of *C. burnetii* harbour the QpH1 plasmid (Dutch strains) [28,38,39]. 

Despite the links between *C. burnetii* genome and virulence factors, there is an urgent need to develop genomic studies to better understand the pathogenicity of this bacterium during pregnancy. 

## 4. The Placenta, a Target Tissue for *C. burnetii*

**Figure 1:**
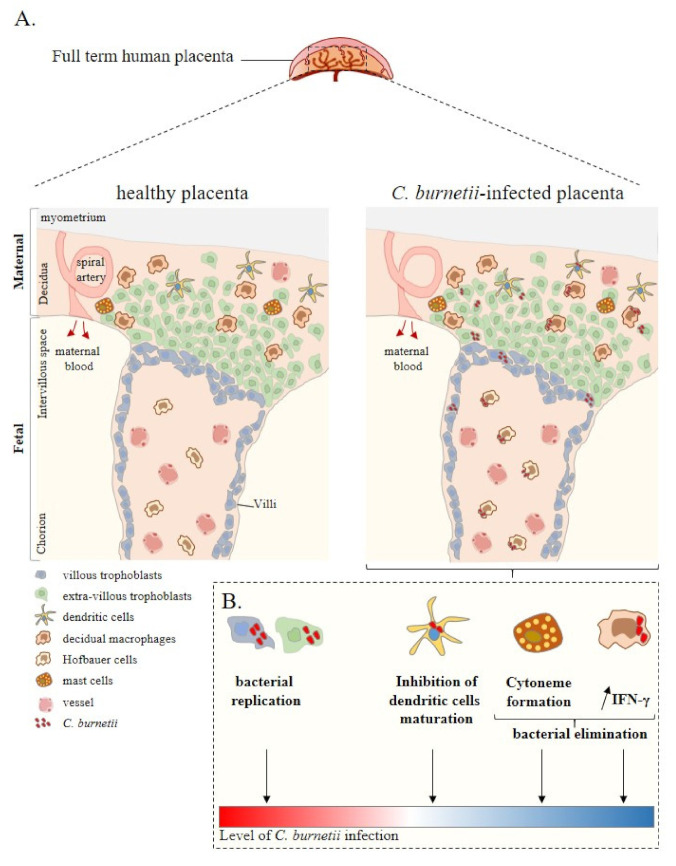
Placenta cell responses against *C. burnetii* infection. (**A**) Schematic representation of a full-term human placenta showing the maternal (decidua, myometrium) and foetal (intervillous space, chorion) parts. Placental cells are represented including extra-villous and villous trophoblasts and immune placental cells such as dendritic cells, macrophages (decidual and Hofbauer), and mast cells. During infection, *C. burnetii* was found in the placental tissue. (**B**) Ex vivo experiments based on in vitro infection of isolated primary cells from healthy at term human placentas reported the infectious capability of trophoblasts, dendritic cells, and macrophages by *C. burnetii*. In this context, although trophoblast infection leads to bacterial replication, the other cell lines present a specific anti-bacterial response promoting bacterial elimination. Thus, ex vivo experiments allow evaluating the level of *C. burnetii* infection at the placental level. Adapted from [42].

The placenta is a complex tissue formed by the chorion and the decidua, corresponding to the foetal and maternal tissues, respectively [38]. Due to its intrinsic structure and plasticity during pregnancy [39], the placenta is essential for foetal growth. Chorionic villus units are composed of mesenchymal cells, decidual macrophages (maternal macrophages), Hofbauer cells (foetal macrophages), foetal vascular cells, and trophoblast cells (Figure 1). The decidua is rich in immune cells, including NK cells, macrophages, T and B lymphocytes, DCs and mast cells, with an over-representation of NK cells and macrophages [40,41]. 

The examination of infected placentas from goats, cows, or mice using immunochemistry reveals the presence of *C. burnetii* organisms in this tissue (Table 1). A direct detection of *C. burnetii* using the polymerase chain reaction technique has confirmed the presence of bacteria in the placental tissue of goats, cows, ewes, calves, and mice [1,21,22,42,43,44]. The bacterium is found at the extracellular level, mainly in necrotic areas, or in placental cells through immunohistochemistry [43,44]. Immunohistochemical investigations show that *C. burnetii* bacteria are found in both the maternal and foetal sections of the placenta of aborted goats (Table 1). 

The anatomopathological examination of animal placentas reveals significant damage to the tissue (Table 1). Inflammatory response and some injury as a consequence are identified in *C. burnetii*-positive placenta from goats, ewes and cows [42,45,47]. Necrosis yellow/brownish exudates are found in *C. burnetii*-positive placentas from abortions and non-abortive pregnancies [5]. Necrotic lesions are observed in chorioallantoic membrane (cotyledonary and intercotyledonary), chorionic epithelium, chorionic villi, and ulcerated chorioallantoic membrane. Leucocyte infiltration is also detected within interplacentomal areas, chorionic and allantoic blood vessel walls at the base of the villi, and in allantochorionic stroma: leucocytes include neutrophils, lymphocytes, and occasional macrophages [5,21,44,45,48]. 

In women infected with *C. burnetii*, two main histological profiles are observed. In symptomatic pregnant women, the placental tissue shows necrosis, villitis, and perivillitis with abundant nuclear debris, necrosis intermixed with numerous disintegrating immune cells, neutrophils, and plasma cells in the decidua [49]. In asymptomatic pregnant women, no foci of necrosis or active inflammation are found in the placenta, but the placental tissue presents fibrotic chorion villi, loss of capillaries, stromal karyorrhexis and haemorrhages [45]. 

## 5. Placental Cells and *C. burnetii* Infection

### 5.1. Trophoblasts

Although the placenta can clearly become infected by *C. burnetii*, it has yet to be determined which types of placental cells are affected by this obligate intracellular bacterium. Based on histological studies, it clearly appears that trophoblasts, which represent the majority of placental cells, are targeted by *C. burnetii* [5,41,45]. Bacteria are found in trophoblasts from the cotyledonary region: the chorioallantoic membrane, intercotyledonary region, chorionic stroma, and chorionic villi (Table 1). *C. burnetii* may be also detected in trophoblasts at the base of the cotyledonary villi [5,41]. It has been suggested that trophoblasts from the chorioallantoic membrane represent the first target of *C. burnetii* before the bacteria spread to adjacent erythrophagocytic trophoblasts in the placentome [46]. 

To dissect the cellular and molecular mechanisms involved in the *C. burnetii* infection of trophoblasts, trophoblastic cell lines may be used. It has been found that *C. burnetii* Nine Mile 1 infects the villous trophoblast BeWo cell line and extra-villous trophoblast JEG-3 cell line, but *C. burnetii* intensively replicates within BeWo cells and is unable to replicate within JEG-3 cells. The mechanism that allows *C. burnetii* to replicate within BeWo cells has been elucidated. Confocal microscopy reveals that the early presence of 27% bacteria in the lysosomal compartment increases to 80% at six days post-infection [50]. The intracellular life cycle of *C. burnetii* Nine Mile 1 in BeWo cell line is based on replication in phagolysosomes. This is distinct from monocyte-derived macrophages in which phagosome maturation is impaired [51]. It may be related to bacterial traffic in non-microbicidal cells in which *C. burnetii* was shown to reside in phagolysosomes or autophagolysosomes. The entry pathways for *C. burnetii* in myeloid cells and trophoblasts may be distinct, leading to distinct intracellular traffic [52]. 

The *C. burnetii* Nine Mile 1-infected BeWo cell line presents a specific transcriptomic program as shown using a whole-genome microarray technique. Of 340 modulated genes, 82% of these modulated genes are up-regulated. The gene ontology (GO) investigation also reveals that modulated genes are involved in apoptosis, cell motility, cell–cell signalling, immune and inflammatory responses. In inflammatory GO terms, modulated genes are organised around the interleukin (IL)-6 and IL-13 networks. Interestingly, genes involved in the development of pregnancy, including early growth response protein-1 (*EGR-1*) and N-Myc downstream regulated 1 (*NDGR1*) genes, are modulated following *C. burnetii* infection [53]. 

### 5.2. Immune Cells

A large amount of placental immune cells suggest that they play an important role in maintaining human pregnancy through the regulation of the local inflammatory environment. The study of the functions of placental immune cells has been mostly investigated in mice. However, murine placentas present important differences in terms of the structure, composition, and function compared to those of humans [54,55], necessitating research with isolated human placental cells. Our team and others have developed specific methods to isolate innate immune cells, including macrophages, DCs, and mast cells using enzymatic digestion, density cushion centrifugation, and positive selection based on specific antibodies [56,57,58,59,60].

#### 5.2.1. Macrophages 

Macrophages represent 20% of the total number of leucocytes found in placenta tissue. During *C. burnetii* infection, macrophages infiltrate placentas of numerous species as shown by immunohistochemistry method (Table 1). To investigate the role of placental macrophages in *C. burnetii* infection, a specific isolation of these cells from healthy at term placentas were realised [56] in order to evaluate their response against Nine Mile bacteria [61]. *C. burnetii* Nine Mile 1 is able to infect placental macrophages in vitro but that bacteria are progressively eliminated until nine days post-infection, as shown by bacterial DNA copy detection and immunofluorescence. Interestingly, it has been previously reported that the spontaneous in vitro fusion of placental macrophages results in the formation of multinucleated giant cells (MGCs) and that placental pathologies such as chorioamnionitis leads to an altered formation of MGCs [62]. When placental macrophages from healthy placentas are infected in vitro by *C. burnetii*, their fusion ability is preserved. Moreover, bacteria are present within MGCs [61]. The role of MGCs remains unclear even in *Mycobacterium tuberculosis* infection, which is the most studied model of infection [63]. 

The key characteristics of macrophages is their plasticity related to their polarisation into inflammatory and microbicidal (also known as M1) or immunoregulatory and non-microbicidal macrophages (also known as M2) according to different microenvironmental stimuli [64]. The proportion of M1/M2 placental macrophages changes during the three trimesters of pregnancy, mainly to maintain a foetal–maternal tolerance [42]. An M1 profile is observed from pre-implantation early in the first trimester, encouraging an environment favourable to the onset of pregnancy. During the second trimester, the M2 profile participates in maintaining a local foetal–maternal tolerance. In the last trimester of pregnancy, the switch towards an M1 profile reflects the process of delivery [65]. Using isolated placental macrophages from healthy at-term placenta, *C. burnetii* infection was found to induce an M1 profile that is different from that induced by LPS, the canonical agonist of M1 polarisation of macrophages, and bacterial elimination [61], while it was previously reported that *C. burnetii* infection leads to an M2 profile in associated monocyte-derived macrophages and persisting infections [66], suggesting a specific role of placental macrophages in the immune defense against pathogens. This M1 profile may be involved in obstetrical complications observed in pregnant women with Q fever, such as spontaneous abortion [67,68] and spontaneous preterm labour [69].

Isolated placental macrophages from healthy at-term placentas spontaneously produce low levels [61] and secrete high amounts of interferon (IFN)-γ during *C. burnetii* Nine Mile 1 infection, although the secretion of IFN-γ by macrophage populations is the subject of significant debate [70]. This inflammatory cytokine is involved in the clearance of intracellular pathogens and is implemented as treatment in response to infection by intracellular pathogens. Interestingly, a significant correlation between in vitro IFN-γ production by placental macrophages and *C. burnetii* elimination was reported, suggesting a key anti-microbial function for IFN-γ [61]. 

#### 5.2.2. Dendritic Cells 

DCs are essential sentinels for the host’s adaptive immune system to detect its environment, especially during infections. At the foetal–maternal interface, uterine DCs play a key role during pregnancy, but this population remains poorly investigated. Isolated placental DCs from healthy at-term placentas do not respond to intracellular bacteria with placental tropism, including *C. burnetii* Nine Mile 1 and *Brucella abortus*. Indeed, decidual DCs stimulated in vitro by *C. burnetii* Nine Mile 1 remain silent in terms of activation and maturation markers studied by flow cytometry (HLA-DR, CD80, CD83, and CD86). Moreover, Shannon et al. reported that in contrast to virulent *C. burnetii* Nine Mile phase I, the infection of monocytes-derived DCs with the avirulent strain led to a Toll-like receptor 4 independent maturation of infected cells [71]. The absence of maturation and activation markers is associated with the inability of decidual DCs to produce inflammatory cytokines [72], suggesting that they play an immunoregulatory role favouring the foetal graft but also *C. burnetii* pathogenicity.

#### 5.2.3. Mast Cells

Mast cells are innate immune cells that serve as sentinels of innate immunity and a regulator of adaptive responses, but they are also involved in allergic reactions [73,74,75]. They are located in various tissues and present both phenotypic and functional plasticities [76]. Placental mast cells from healthy at-term placentas are involved in the regulation of tissue remodelling, angiogenesis, and immune response during infections [77]. Following in vitro *C. burnetii* Nine Mile 1 simulation of placental mast cells, CD36 and Toll-like receptor 4, which are involved in bacterial recognition, play a role in the formation of cytonemes, extracellular actin filaments. The expression of antimicrobial peptides (cathelicidin and neutrophil elastase) on cytonemes suggests that cytonemes play an important role in the destruction of entrapped bacteria before their entry into host cells [78]. 

## 6. Conclusions

To date, the analytical approach of the response of each type of placental cells to *C. burnetii* infection has contributed to a better understanding of the placental response to this pathogen. However, this approach is insufficient to study the complexity, plasticity, and specific properties of the placenta. A more integrated approach may consist in the study of cell populations in infected human placenta by single cell sequencing [79], culture of each placental cell type with supernatants of the other cell types, cell co-culture, or ex vivo perfusion of the placenta and to study the modulation of *C. burnetii* infection. Combining these approaches with the analysis of placentas from women infected with *C. burnetii* may allow for a better understanding of both the normal functioning of placentas and their dysfunctions observed during infections such as with *C. burnetii*. 

## Figures and Tables

**Table 1:** Placenta histology and *C. burnetii* infection.

Species	Placenta Histology and Cellular infiltration		*Coxiella burnetii* Presence in Placenta	References
Human	Symptomatic woman	Maternal part: Necrosis intermixed with disintegrating immune cells, neutrophils and plasma cells Foetal part: Necrosis on villitis and perivillitis (nuclear debris)	Placenta positive for *C. burnetii*	[36,45]
	Asymptomatic woman	Maternal part: No foci of necrosis or active inflammation Foetal part: Fibrotic chorion villi, loss of capillaries, stromal karyorrhexis and haemorrhages	Not reported	
Goat	Maternal part: Necrosis (severe placentitis) with neutrophil infiltration (chorionic area) Ulcerated trophoblasts of the chorioallantoic membrane Infiltration of neutrophils, lymphocytes and occasionally of macrophages Allantochorion: necropsys and yellow/brownish exudate Foetal part: Necrosis of the chorionic epithelium and placentitis Placenta vasculitis Ulceration of chorionic villi and suppurative inflammation Inflammation of the cotyledon Trophoblast layer with necropurulent inflammation and dystrophic calcification Inflammatory exudate of neutrophils, eosinophils, lymphocytes and macrophages		Maternal part: Trophoblasts positive for *C. burnetii* Extracellular presence of *C. burnetii* in necrotic areas Endometrium and stroma negatives for *C. burnetii* Foetal part: Cotyledons positive for *C. burnetii* Increased number of trophoblasts in cotyledons Trophoblasts negative for *C. burnetii*	[5,22,46]
Cow	Maternal part: Infiltration of mononuclear cells in the stroma Foetal part: Chorionic villi: necrosis and neutrophil exudation. Swollen trophoblasts Entire placenta: Necrotic trophoblasts Stromal infiltration and oedema Necrotising placentitis Vasculitis and inflammation Infiltration of neutrophils, lymphocytes, macrophages and necrotic trophoblasts Cytoplasmic granules within swollen trophoblasts		Cotyledonary trophoblasts	[40,43,45,47]
Mouse	Maternal part: Diffuse necrosis Infiltration of neutrophils, monocytes and macrophages in the decidua basalis Foetal part: Labyrinth: inflammatory lesions, necrosis and infiltration of neutrophils and macrophages Chorionic: single-cell necrosis		Not reported	[48]

## Data Availability

No new data were created or analyzed in this study. Data sharing is not applicable to this article.
